# Relationships of Cuproptosis-Related Genes With Clinical Outcomes and the Tumour Immune Microenvironment in Hepatocellular Carcinoma

**DOI:** 10.3389/pore.2022.1610558

**Published:** 2022-09-21

**Authors:** Xi Chen, Gang Hu, Li Xiong, Qingqing Xu

**Affiliations:** ^1^ Department of Thoracic Oncology, Huangshi Central Hospital, Affiliated Hospital of Hubei Polytechnic University, Edong Healthcare Group, Huangshi, China; ^2^ Department of Breast Surgery, Thyroid Surgery, Huangshi Central Hospital, Affiliated Hospital of Hubei Polytechnic University, Edong Healthcare Group, Huangshi, China; ^3^ Department of Radiology, Huangshi Central Hospital, Affiliated Hospital of Hubei Polytechnic University, Edong Healthcare Group, Huangshi, China; ^4^ Department of Pathology, Huangshi Central Hospital, Affiliated Hospital of Hubei Polytechnic University, Edong Healthcare Group, Huangshi, China

**Keywords:** immunotherapy, hepatocellular carcinoma, immune infiltration, prognostic model, cuproptosis

## Abstract

**Background:** Cuproptosis is a recently identified form of regulated cell death that plays a critical role in the onset and progression of various cancers. However, the effects of cuproptosis-related genes (CRGs) on hepatocellular carcinoma (HCC) are poorly understood. This study aimed to identify the cuproptosis subtypes and established a novel prognostic signature of HCC.

**Methods:** We collected gene expression data and clinical outcomes from the TCGA, ICGC, and GEO datasets, analysed and identified 16 CRGs and the different subtypes of cuproptosis related to overall survival (OS), and further examined the differences in prognosis and immune infiltration among the subtypes. Subtypes-related differentially expressed genes (DEGs) were employed to build a prognostic signature. The relationship of the signature with the immune landscape as well as the sensitivity to different therapies was explored. Moreover, a nomogram was constructed to predict the outcome based on different clinicopathological characteristics.

**Results:** Three cuproptosis subtypes were identified on the basis of 16 CRGs, and subtype B had an advanced clinical stage and worse OS. The immune response and function in subtype B were significantly suppressed, which may be an important reason for its poor prognosis. Based on the DEGs among the three subtypes, a prognostic model of five CRGs was constructed in the training set, and its predictive ability was validated in two external validation sets. HCC patients were classified into high and low-risk subgroups according to the risk score, and found that patients in the low-risk group showed significantly higher survival possibilities than those in the high-risk group (*p* < 0.001). The independent predictive performance of the risk score was assessed and verified by multivariate Cox regression analysis (*p* < 0.001). We further created an accurate nomogram to improve the clinical applicability of the risk score, showing good predictive ability and calibration. Low- and high-risk patients exhibit distinct immune cell infiltration and immune checkpoint changes. By further analyzing the risk score, patients in the high-risk group were found to be resistant to immunotherapy and a variety of chemotherapy drugs.

**Conclusion:** Our study identified three cuproptosis subtypes and established a novel prognostic model that provides new insights into HCC subtype prognostic assessment and guides more effective treatment regimens.

## Introduction

Hepatocellular carcinoma (HCC) comprises approximately 90% of primary liver cancers in the world. It is the fifth most prevalent cancer and ranks fourth among cancer-related deaths worldwide [1]. HCC causes approximately 800,000 deaths each year and seems to have a heavy disease burden [2]. In the past 2 or 3 years, there have been some notable advances in the treatment of HCC, such as resection and transplantation [3, 4]. Recently, immunotherapy and molecular targeted therapy for HCC have also been developed and are expected to become new treatment approaches [5,6,7]. However, the survival rate of HCC is still far from satisfactory due to a low rate of early detection, a tendency for recurrence, and chemotherapy resistance [8]. Therefore, it is of great significance for us to identify accurate biomarkers in the diagnosis stage of patients with HCC to evaluate the prognosis of HCC.

Regulated cell death (RCD) is the primary mechanism for eliminating damaged, infected, or redundant cells [9, 10]. Apoptosis was originally thought to be the only RCD mechanism. Nevertheless, as the understanding of the cellular mechanisms that mediate RCD continues to grow, many new forms of non-apoptotic RCD have been discovered, including ferroptosis, pyroptosis, necroptosis, and autophagic cell death [9, 11, 10]. In 2012, Dixon et al. [12] defined the term ferroptosis to describe the form of cell death induced by the small molecule Erastin, which is iron ion-catalyzed necrotic cell death by inhibiting cystine import, resulting in glutathione depletion and phospholipid peroxidase glutathione peroxidase inactivation 4 (GPX4). Ferroptosis was related to the pathophysiological changes of many cancers [13, 14]. Triggering ferroptosis as a novel approach to cancer treatment is highly anticipated and an active area of research. Pyroptosis is an inflammatory RCD that is mainly triggered by inflammatory caspases and gasdermin family proteins and is manifested by the continuous swelling of cells until cell membrane rupture and death [15].

Interestingly, a recent study by Tsvetkov and others revealed that intracellular copper induces a new form of RCD distinct from oxidative stress-related cell death, known as “cuproptosis” [16]. It occurs through the direct binding of copper to fatty acylated components of the tricarboxylic acid cycle, resulting in fatty acylated protein aggregation and iron-sulphur cluster protein loss, leading to proteotoxic stress and ultimately cell death [16]. Studies have shown that an imbalance in copper homeostasis affects tumour growth, causing irreversible damage. Copper can induce multiple forms of cell death through various mechanisms, including reactive oxygen species accumulation, proteasome inhibition, and anti-angiogenesis [17]. For example, blocking SLC31A1-dependent copper uptake increases autophagy in pancreatic cancer cells against cell death [18]. At present, several genes and proteins have been shown to regulate cuproptosis, including FDX1, LIAS, LIPT1, PDHA1, and PDHB [16]. However, the expression patterns and clinical value of cuproptosis-related genes (CRGs) in HCC remain unclear.

In this study, we firstly built a predictive signature based on CRGs as a prognostic biomarker. Next, we created an accurate nomogram to improve the clinical applicability of the risk score. In addition, we analyzed the correlation of CRGs with the prognosis, the TME, immune checkpoint genes, chemotherapy sensitivity, and immunotherapy.

## Materials and Methods

### Data Source

The RNA-seq data and clinical traits for HCC patients were obtained and extracted from the TCGA, ICGC, and GEO (GSE14520) datasets. Among them, TCGA contained 371 samples, GSE14520 contained 242 samples, and ICGC contained 260 samples. The “sva” and “limma” R packages were implemented to integrate and normalize the RNA-seq data and microarrays separately. The data of 265 HCC patients with complete clinical information and follow-up time in TCGA were used as the training set to build a prognostic model related to cuproptosis, and the GSE14520 dataset (221 patients) and ICGC dataset (232 patients) were used as two external validation sets. Additionally, 16 CRGs (PDHA1, DLD, DLAT, PDHB, GLS, MTF1, SLC31A1, CDKN2A, LIPT1, FDX1, LIAS, ATP7A, ATP7B, BAD, CCS, MTOR, and NRF2) used in this study were obtained from previous publications [16,18–24].

### Unsupervised Consensus Clustering of the HCC Molecular Subtypes

With the “ConsensusClusterPlus” R package, a consensus clustering method was applied to categorize patients into different molecular subtypes on the basis of the CRG expression levels. The optimal number of subtypes k was identified by considering where the magnitude of the cophenetic correlation coefficient decreased. Subsequently, the relationship between molecular subtypes and clinicopathological features and prognosis were compared.

### Immune Landscape of the Molecular Subtypes

We explored the difference of each subtype in the TME score using the ESTIMATE algorithm. In addition, the CIBERSORT algorithm was used to predict HCC samples’ immune-infiltrating cells. Upon entering the samples’ expression data, we obtained the sample’s proportion of 22 immune-infiltrating cells.

### Construction and Validation of the Cuproptosis-Related Gene Predictive Signature

The “limma” R package was employed to screen the differentially expressed genes (DEGs) between different subtypes according to the following criteria: |fold change| > 1.5 and *p* < 0.05. We used a univariate Cox analysis based on DEGs to assess the prognostic significance of candidate DEGs in HCC. After further adjustment, multivariate Cox regression (stepwise model) was performed to identify the pivotal genes, which were employed to build prognostic signature. The coefficients obtained from the regression algorithm were used to obtain the risk scores based on the following formula:

Risk score=ExpGene1∗β1+ExpGene2∗β2+...+ExpGenen∗βn

Furthermore, the patients were classified into two risk groups (high and low) using the median as the cut-off value. Kaplan-Meier survival curves were generated to assess differences between the two risk groups. A ROC curve was employed to evaluate the performance of the model. Principal component analysis (PCA) was conducted using the “prcomp” function of the “stats” R package to explore the distribution of different groups.

We chose two datasets, GSE14520 and ICGC, as the external verification set to verify the predictive accuracy of the signature. Patients were stratified into high- and low-risk groups based on the cut-off point of the risk score of the TCGA set. Kaplan–Meier survival curves were generated to assess the differences between the two risk groups. ROC curves and PCA were employed to evaluate the validity of the prognostic signature.

### Prognostic Value of the Risk Model

We analysed the association of the risk scores with clinicopathological traits, including age, sex, grade, pathologic stage, and surgical procedure. To determine whether the signature was an independent prognostic indicator, we performed univariate and multivariate Cox analyses.

### Nomogram Construction and Assessment

The multivariate Cox regression analysis of clinical parameters and risk score were utilized to build a prognostic nomogram using the “rms” package. The ROC curve was ploted to assess the predictive accuracy of the nomogram. Decision curve analysis (DCA) was used to assess the clinical benefits and utility of the nomogram. Afterward, calibration plots were developed to evaluate the correlation between the actual and predicted survival.

### Tumour Immune Microenvironment Analysis

To clarify the potential regulatory role of the signature in immune cell infiltration, we explored the infiltration of 22 immune cells in the low- and high-risk groups. Considering the importance of immune checkpoint-related gene expression levels for immune checkpoint inhibitor therapy, we assessed the relationship between the risk score and immune checkpoint expression in HCC patients. Meanwhile, the potential response of HCC samples to immunotherapy was inferred by the TIDE algorithm [25].

### Investigation of Differences in the Chemotherapeutic Efficacy

To assess the significance of the predictive signature in predicting the sensitivity to chemotherapy in HCC, the “pRRophetic” package was used to calculate the IC50 of the main chemotherapeutic medications used in the treatment of HCC patients.

### Functional Enrichment Analysis

To explore the signalling pathways in which the cuproptosis-related signature may be involved in regulation, DEGs between the two risk score subgroups were retrieved (|log_2_FC| ≥ 2 and adjusted *p* < 0.01) for GO and Kyoto Encyclopedia of Genes and Genomes (KEGG) analyses with “cluster Profiler” in R.

## Results

### Unsupervised Consensus Clustering of the HCC Molecular Subtypes

**FIGURE 1:**
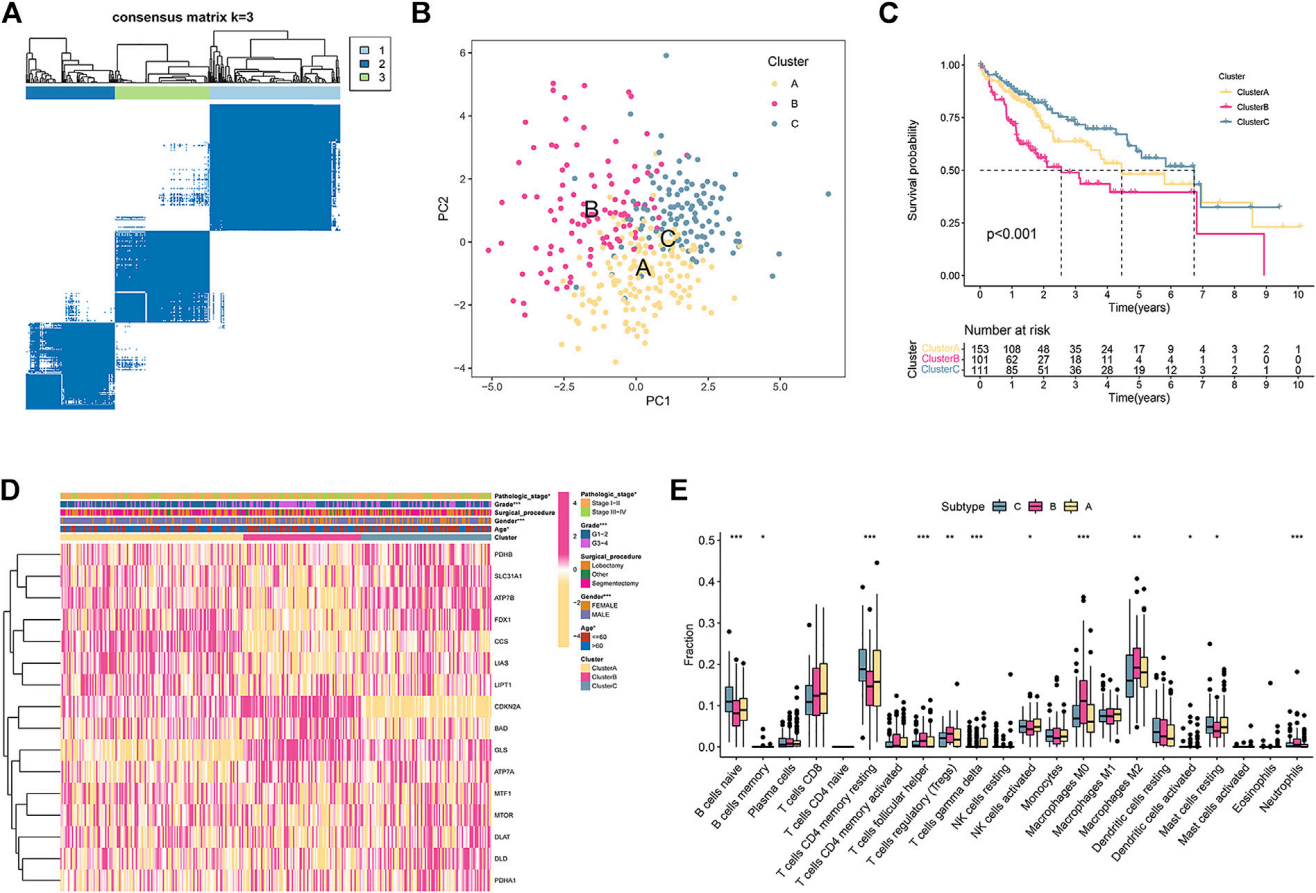
HCC subtypes according to cuproptosis-related genes. **(A)** The consensus score matrix of all samples in the TCGA set at k = 3. **(B)** The PCA distribution plot distinguished three subtypes. **(C)** Kaplan–Meier curve of the three subtypes. **(D)** Heatmap of clinical traits and cuproptosis-related gene expression in the three subtypes. **(E)** The enrichment scores of 22 immune cells in the three subtypes.

Based on the expression of 16 CRGs, a consensus clustering method was carried out in the TCGA cohort. At a clustering variable (k) of 3, the intragroup and intergroup correlations were high and low, respectively, suggesting that the 371 HCC patients clustered into three groups (Figure 1A). PCA demonstrated that the individuals were distributed into three delineated clusters (Figure 1B). Kaplan-Meier survival curves revealed that patients in subtype B had a markedly shorter OS than those in subtypes A and C (*p* < 0.001; Figure 1C). The heatmap displayed the distribution of the clinicopathological traits and CRG expression among the three subtypes (Figure 1D). Most CRGs had lower expression levels in subtype B than in the other subtypes (Figure 1D). Patients in subtype B were elderly, had a higher grade, and had an advanced pathologic stage (Figure 1D).

### Immune Landscape of the Molecular Subtypes

We further examined whether any differences were observed regarding TIICs to investigate the immunological characteristics of HCC. The CIBERSORT algorithms were used to explore the associations among TIICs and the three subtypes. The fraction of memory and resting memory CD4 T cells, resting mast cells, naive B cells, activated dendritic cells, and activated NK cells were significantly downregulated in subtype B (*p* < 0.05; Figure 1E). In contrast, helper follicular cells, Tregs, neutrophils, and M0 and M2 macrophages were markedly upregulated in subtype B (*p* < 0.05).

### Construction and Validation of the Cuproptosis-Related Gene Predictive Signature

**FIGURE 2:**
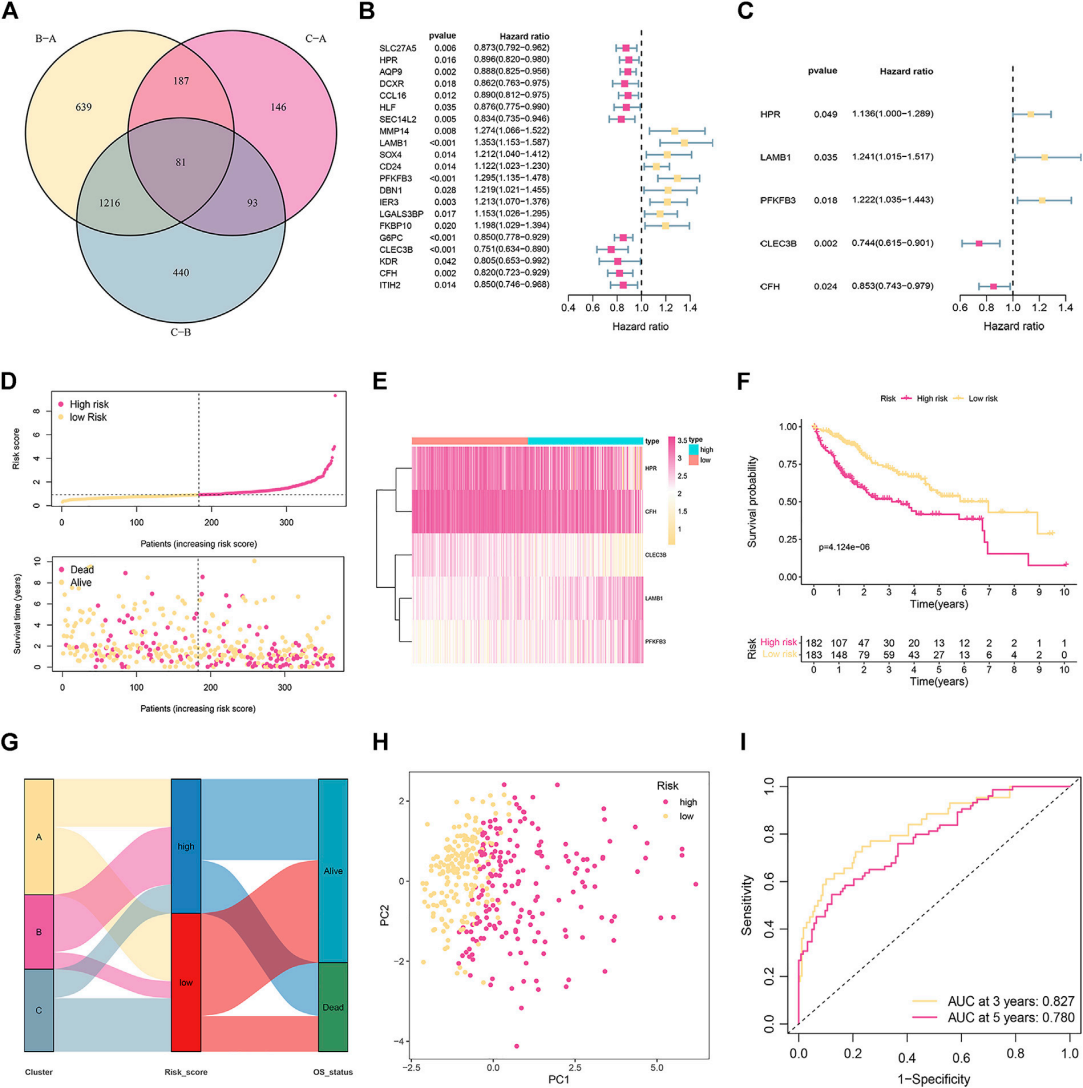
Establishment and validation of the prognostic signature in the training cohort. **(A)** Venn diagram to identify differentially expressed genes (DEGs) among the different subtypes. **(B)** Univariate Cox regression analysis of 21 DEGs. **(C)** The presentation of five independent prognostic genes in multivariate Cox regression analysis. **(D)** Risk score distribution and survival status of patients. **(E)** The relative expression of the five genes in different risk groups. **(F)** Kaplan-Meier curves of OS in different risk groups. **(G)** The distribution of cuproptosis subtypes, risk score, and survival status. **(H)** PCA displays an obvious difference in transcriptomes between the two risk groups. **(I)** ROC curve of survival rate for the signature.

Based on the criteria of *p* < 0.05 and |FC| > 1.5, and 81 subtype-related DEGs were identified (Figure 2A). Then, 21 prognosis-related DEGs were found significantly correlated with the OS of HCC patients according to univariate Cox regression analysis (*p* < 0.05; Figure 2B). Furthermore, we conducted multivariant Cox regression analysis on these 21 genes. According to the Akaike information criterion (AIC) value, we finally obtained five genes to construct risk models, including HPR, LAMB1, PFKFB3, CLEC3B, and CFH (Figure 2C). Afterward, we computed the risk score as follows:

$$\begin{array}{l} Risk\;score=0.1273\times expression\left(HPR\right)+0.2161\times expression\left(LAMB1\right)+0.2006\times expression\left(PFKFB3\right)-0.2951\times expression\left(CLEC3B\right)\\ -0.1589\times expression\left(CFH\right). \end{array}$$

The median risk score was used as the cut-off to categorize the HCC patients into two groups: low-risk (*n* = 183) and high-risk (*n* = 182). Figure 2D represents the status of survival and the distribution of the risk scores, whereas Figure 2E shows the relative expression of the 5 genes for each patient in the two groups. According to the Kaplan-Meier plot, patients with high risk showed a considerably lower OS (Figure 2F). The distribution of cuproptosis subtypes, risk score, and survival status are shown in Figure 2G. PCA could divide patients with different risks into two groups (Figure 2H). ROC evaluated the prediction performance of the signature. The AUC showed that the 3- and 5-year OS were 0.827 and 0.780, respectively (Figure 2I).

**FIGURE 3:**
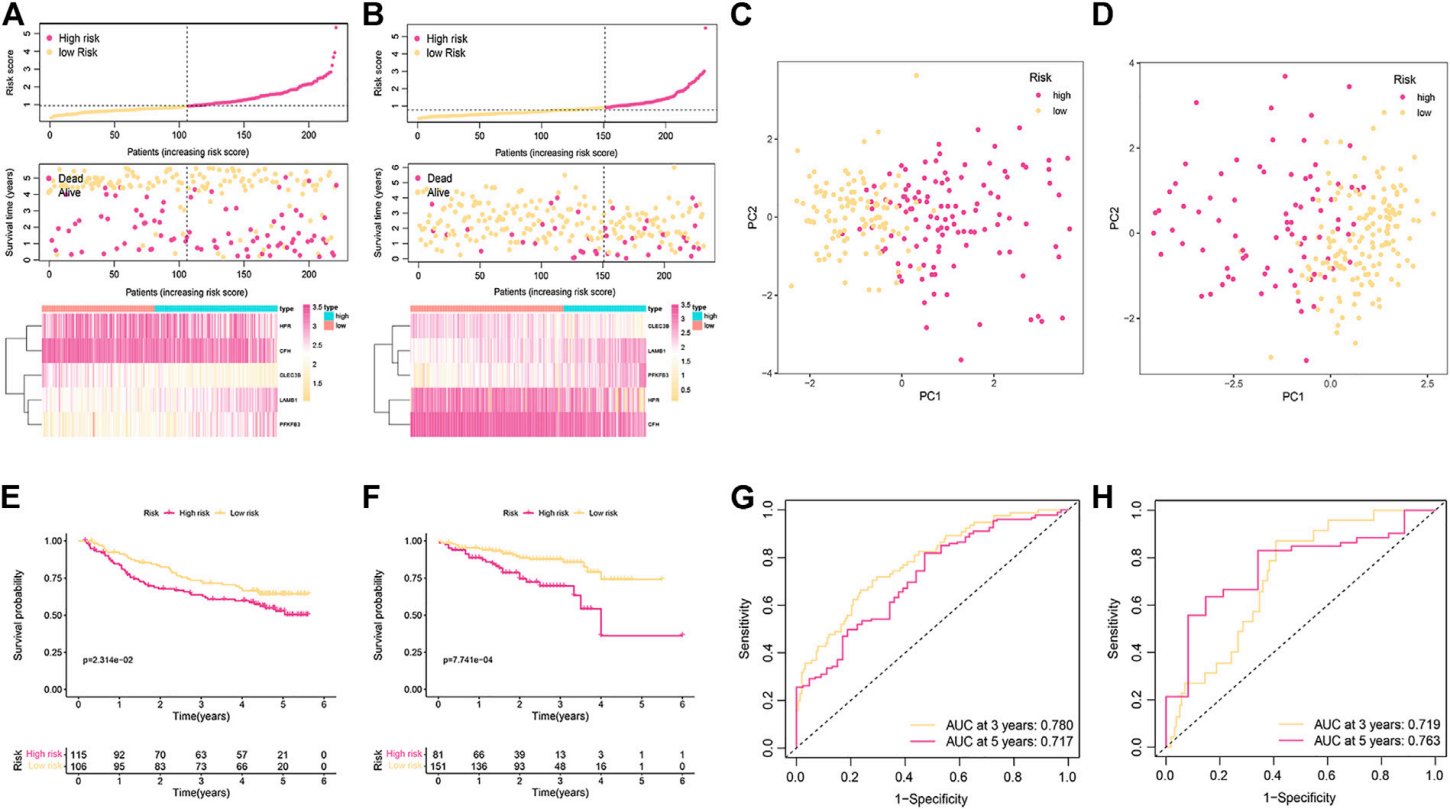
Validation of the cuproptosis-related signature in the GSE14520 and ICGC cohorts. **(A,B)** The distribution of risk scores and survival status of patients in the GSE14520 **(A)** and ICGC cohorts **(B)**. **(C,D)** PCA plot for two subgroups in the GSE14520 **(C)** and ICGC cohorts **(D) (E,F)** Kaplan-Meier curves of OS in the GSE14520 **(E)** and ICGC cohorts **(F)**. **(G,H)** ROC curve for assessing the prognostic value of the signature in the GSE14520 **(G)** and ICGC cohorts **(H)**.

The reliability of the signature related to cuproptosis was verified by two external validation cohorts (GSE14520 and ICGC). The risk score was established by using the previous formula. The status of survival and the distribution of the risk scores, and the relative expression of the five genes for each patient in each of the two groups are shown in Figures 3A,B. PCA could divide patients with different risks into two clusters (Figures 3C,D). Low-risk patients had favorable OS compared with their high-risk counterparts (Figures 3E,F). The ROC curve demonstrated that the signature had a favorable predictive performance (Figures 3G,H).

### Prognostic Value of the Risk Model

**FIGURE 4:**
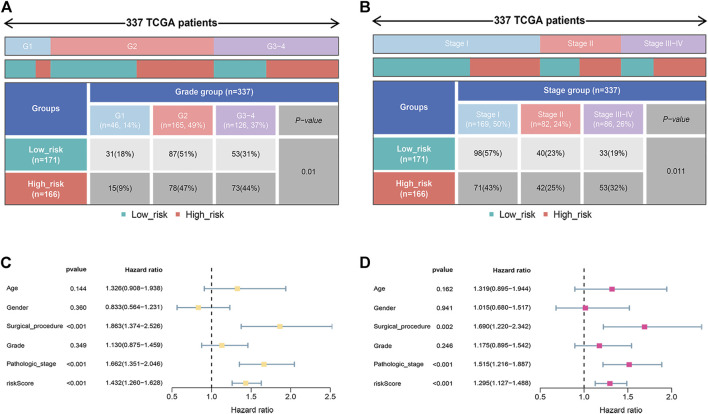
Prognostic value of the signature. **(A)** The relationship between the risk score and tumor grade. **(B)** The relationship between the risk score and pathologic stage. **(C,D)** Univariate and multivariate analyses indicated the prognostic value of the risk score.

The correlation between the risk score and clinicopathological traits was further analyzed. As shown in Figures 4A,B, the risk score was correlated with grade (*p* = 0.01) and pathologic stage (*p* = 0.011). To further confirm the independence of the model, we performed univariate and multivariate Cox analyses. As shown in Figures 4C,D, the risk score, surgical procedure, and pathologic stage were independent prognostic factors.

### Nomogram Construction and Assessment

**FIGURE 5:**
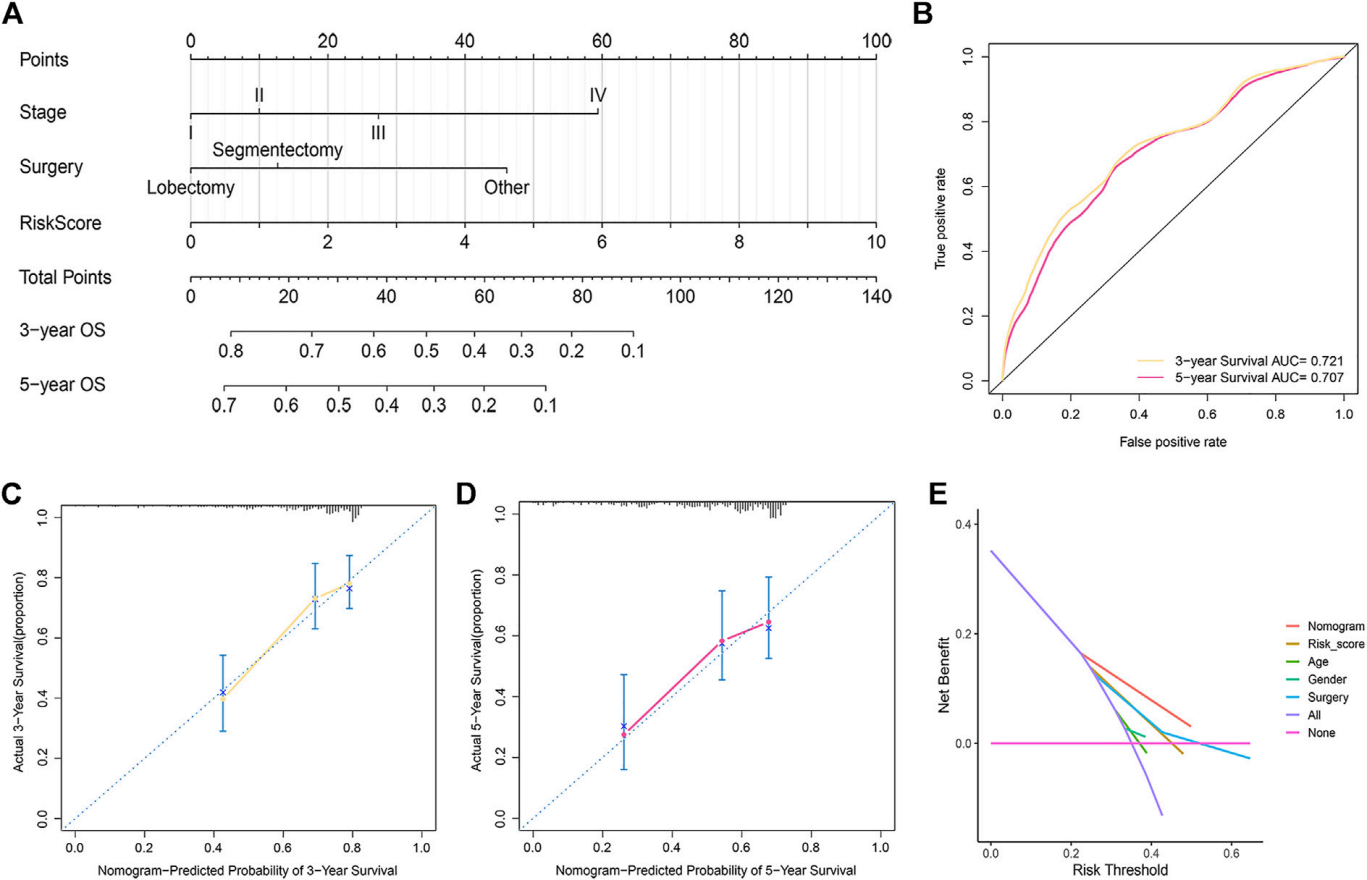
Development and assessment of the prognostic nomogram. **(A)** The nomogram that integrated the risk score, stage, and surgery predicted the probability of the 3- and 5-year OS. **(B)** The 3- and 5-year time-dependent ROC curves. **(C,D)** The calibration curves for 3- **(C)** and 5-year OS **(D)**. **(E)** Decision curve analysis of the nomogram, risk score, age, sex, grade, surgical procedure, and pathologic stage.

Based on the above results, a nomogram was built to forecast the survival risk in HCC patients. The nomogram, which is based on risk score, surgical procedure, and pathologic stage, can predict the three- and five-year OS (Figure 5A). In order to assess the sensitivity and specificity of the nomogram on the prognosis, the ROC was performed. The nomogram displayed AUC values of 0.721 and 0.707 at 3- and 5-year in the ROC analysis, respectively (Figure 5B). We also used the 3- and 5-year calibration plots to prove that the proposed nomogram had a similar performance compared to an ideal model (Figures 5C,D). Furthermore, the DCA showed better clinical benefit and utility of nomogram for predicting OS (Figure 5E).

### Tumour Immune Microenvironment Analysis

**FIGURE 6:**
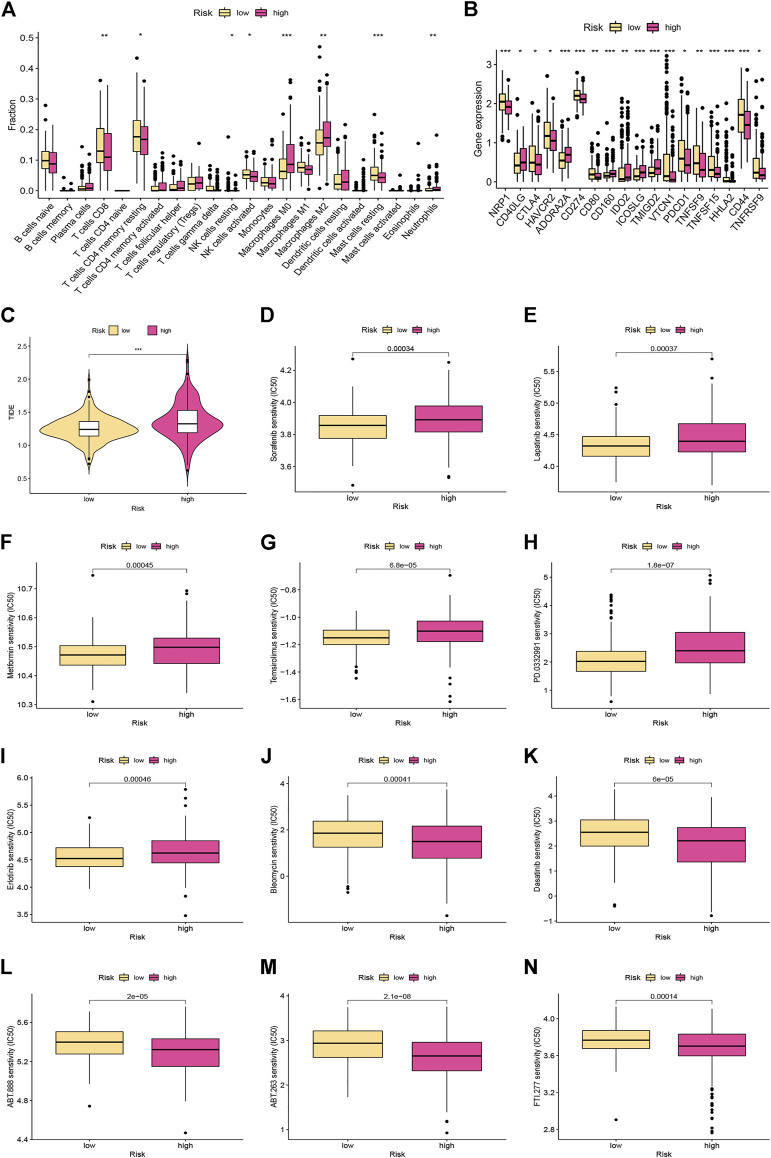
Immune landscape and drug sensitivity analyses between the low- and high-risk groups. **(A)** The proportion of 22 immune infiltrating cells in the low- and high-risk groups. **(B)** The expression levels of the immune checkpoints in different risk subgroups. **(C)** Comparison of the TIDE scores between the different risk groups. **(D–N)** Chemotherapeutic sensitivity of patients in the different risk subgroups.

We performed CIBERSORT algorithms for different immune cell subsets, to further study the relationship between risk score and immune status in the two subgroups. As shown in Figure 6A, the high-risk group had lower levels of infiltration in a variety of immune cells, including resting memory CD4 T cells, CD8 T cells, resting NK cells, activated NK cells, and resting mast cells. In contrast, the high-risk group had higher infiltration of M0 and M2 macrophages. We further investigated the potential role of the signature in assessing the immunotherapy efficacy of ICIs in HCC patients by analyzing the association between the signature and prevalent ICI targets. Patients in the low-risk group had higher expression of these genes (e.g., CD274, CTLA4, HAVCR2, and PDCD-1) in comparison to the high-risk group (Figure 6B). In terms of immunotherapy, we explored the responses of HCC samples to immunotherapy. Compared with HCC patients with high-risk score, patients with low-risk score were more sensitive to immunotherapy (Figure 6C).

### Investigation of Differences in Chemotherapeutic Efficacy

Distinct HCC subgroups should be used to guide clinical treatment. Correlation between the risk score and the sensitivity to chemotherapy was investigated. The results indicated that the IC_50_ values of sorafenib, lapatinib, metformin, temsirolimus, palbociclib (PD-0332991), and erlotinib were significantly lower in samples of the low-risk group, while the IC_50_ values of bleomycin, dasatinib, veliparib (ABT-888), ABT-263, and FTI-277 were significantly lower in samples of the high-risk group (Figures 6D–N).

### Functional Enrichment Analysis

**FIGURE 7:**
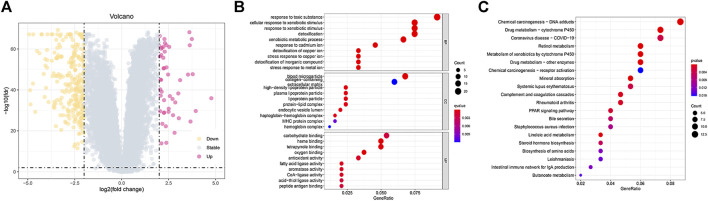
Functional enrichment analysis between the low- and high-risk groups. **(A)** Volcano plot of 334 DEGs between the different risk groups. **(B,C)** GO and KEGG enrichment analyses of DEGs among the two risk groups.

According to the criteria for FDR < 0.01 and |FC| > 1.5, we identified 334 DEGs in HCC samples (Figure 7A). According to GO analysis, the biological processes of DEGs were primarily enriched in response to a toxic substance, detoxification of copper ion, and stress response to copper ion (Figure 7B). The cellular components of the DEGs were mainly enriched in blood microparticles, high-density lipoprotein particles, and endocytic vesicle lumen (Figure 7B). The molecular functions of DEGs were mainly enriched in oxygen binding, haem binding, and tetrapyrrole binding (Figure 7B). KEGG pathway analyses revealed that these genes were primarily enriched in chemical carcinogenesis-DNA adducts, drug metabolism-cytochrome P450, linoleic acid metabolism, and drug metabolism-other enzymes (Figure 7C).

## Discussion

After developing chronic fibrotic liver disease caused by viral or metabolic aetiologies, patients tend to progress to HCC [26]. Nevertheless, the key issue at present is that there is no robust estimation system that can reliably estimate the HCC risk or diagnose HCC during its early stages [27]. As a result, identifying biomarkers is critical for early detection, prognostic analysis, and individualized therapy of HCC.

RCD, or, more specifically, cell suicide, is not only essential in embryonic development but also plays a critical role in the occurrence and development of diseases, especially malignancies [11]. Apoptosis is one of the most classic forms of programmed cell death and is considered the most promising target for tumor therapy [28]. In addition to classical apoptosis, several other forms of RCD have been identified [11]. Copper ionophore-induced cell death is a novel cell death pathway that is distinctly different from traditional death methods [16]. Cuproptosis plays an important role in tumorigenesis and cancer therapy [18, 16, 29]. Dysregulation of CRGs has been shown to be involved in the pathogenesis and development of multiple types of cancer. Li et al. [30] showed that copper chaperone for superoxide dismutase (CCS) promotes breast cancer cell growth and migration by regulating ROS-mediated ERK1/2 activity. Vyas et al. [31] reveal that copper-dependent ATP7B upregulation drives resistance to platinum toxicity in a TMEM16A-overexpressing head and neck cancer model.

Physiologically, enterocytes take up bioavailable copper ions from the diet in a Ctr1-dependent manner, and upon incorporation, cuprous ions are transported to ATP7A, which pumps Cu^+^ from enterocytes into the blood. Copper ions reach the liver through the portal vein and enter hepatocytes through Ctr1 to form membrane pores. Then, Cu^+^ can be secreted into bile or blood through the Atox1/ATP7B/ceruloplasmin pathway. In the blood, this micronutrient can reach peripheral tissues and be reabsorbed by Ctr1 [32]. Wilson’s disease (WD) is an autosomal recessive disorder caused by mutations in the ATP7B gene, which encodes the copper-transporting ATPase, resulting in impaired hepatic copper excretion [33]. Copper metabolism must be tightly controlled in order to achieve homeostasis and avoid disorders. A hereditary or acquired copper unbalance may cause or aggravate many diseases, including cardiovascular diseases, neurodegenerative diseases, Genetic disorders, metabolic diseases, and cancer [34, 35].

The main risk factors for HCC are hepatotropic viruses (HBV and HCV) [36]. In addition, nonalcoholic fatty liver disease (NAFLD) is already the fastest growing cause of HCC in the USA [37]. Evidence reports that inadequate copper intake serum concentration is involved in the pathogenesis of NAFLD. Intrahepatic and serum copper concentrations were lower in subjects with NAFLD compared with other liver diseases [38]. NAFLD-cirrhotic patients were characterized by a statistical significant enhancement of serum copper levels, even more evident in HCC patients [39, 38]. Patients with hepatolenticular degeneration due to impaired copper metabolism have a high incidence of HCC [40], so we hypothesize that cuproptosis has a correlation with the development of HCC. However, the mechanism of action of cuproptosis affecting HCC is not clear.

RCD-based HCC subtype and/or prognostic models are becoming a research hotspot for predicting HCC prognosis. Growing evidence suggests that prognostic models based on next-generation sequencing and public databases provide more comprehensive clinical-genetic prognostic value [41,42,43]. The role including clinical relevance and prognostic significance of CRGs in HCC is unknown. In the present study, TCGA and two external databases (GEO and ICGC) were employed to collect gene expression and clinicopathological information as the training and validation cohorts, respectively. According to an unsupervised consensus clustering analysis of 16 CRGs, HCC patients were categorized into three subtypes. Compared with patients with other subtypes, patients with subtype B had worse OS. The level of immune cell infiltration also differed greatly among the subtypes. The contents of infiltrated immune cells in subtype A and C were significantly higher than those in subtype B, which seemed to contradict its worse survival outcome.

To better evaluate each HCC patient’s prognosis and therapeutic response, it is essential to construct a cuproptosis signature, which could generate a cuproptosis model for individual prediction. Therefore, we identified 81 cuproptosis subtype-related DEGs and developed and tested a novel prognostic signature in HCC patients by using the identified DEGs. The signature contained five cuproptosis-associated genes: PFKFB3, CLEC3B, CFH, HPR, and LAMB1. PFKFB3, a key molecule in glucose metabolism in the cytoplasm, obviously accelerates the rate of glycolysis and is expressed in rapidly proliferating cells and multiple cancers [44]. PFKFB3 can promote cell cycle progression and inhibit apoptosis through Cdk1-mediated phosphorylation of p27, while MAPK increases PFKFB3 transcripts to accelerate cell proliferation [44]. PFKFB3 has been shown to affect the tumorigenesis and progression of HCC through various mechanisms and is a potential target for the treatment of HCC [44–47]. Li et al. [44] found markedly increased OS in individuals with PFKFB3-overexpressing tumors in comparison with the low-expression group. Knockdown of PFKFB3 reduces glucose consumption and disrupts DNA repair function, resulting in G2/M phase arrest and apoptosis in HCC cells. Mechanistically, blockade of PFKFB3 inhibits hepatocellular carcinoma growth by impairing DNA repair via AKT. Matsumoto et al. [46] revealed that inhibition of PFKFB3 suppressed tumor growth and induced tumor vascular normalization in HCC. CLEC3B, a member of the C-type lectin superfamily, has been reported to be downregulated in serum and tumor tissues of HCC [48, 49]. Dai et al. [48] found that downregulation of exosomal CLEC3B in HCC promoted metastasis and angiogenesis through AMPK and VEGF signaling. CFH is a critical regulatory protein of the alternative complement pathway. Mao et al. [50] showed that CFH is enriched in extracellular vesicles (EVs) of metastatic HCC cells and it protects HCC cells by evading complement attack, thereby promoting HCC progression. Therefore, the expression of CRGs was strongly associated with the tumorigenesis, invasion, and outcomes of hepatocellular cancer, corroborating our findings.

The HCC patients were then divided into two risk subgroups based on the calculated cut-off point. Kaplan-Meier curves indicated that the OS rate was markedly higher in the low-risk group than in the high-risk group. The time-dependent ROC curve showed that the risk score presented a good performance for survival prediction. External validation confirmed the value of the predictive signature. Furthermore, multivariate Cox regression analysis confirmed that the risk score was a prognostic factor independent of clinical characteristics. We then developed a nomogram for predicting 3- and 5-year OS in HCC patients, and we also verified the accuracy of the nomogram by calibration. Taken together, the signature may be effective in predicting patient outcomes, thereby facilitating the implementation and evaluation of the model in future clinical practice.

The tumor immune microenvironment is crucial in the initiation and progression of HCC [51]. We calculated 22 TIICs in the two risk subgroups according to the CIBERSORT algorithm. We observed that risk scores were negatively correlated with resting memory CD4 T cells, CD8 T cells, resting and activated NK cells, whereas risk scores were positively correlated M0 and M2 macrophages, indicating that the signature may significantly contribute to modulating immune cell infiltration. Emerging experiments and clinical studies have found that immunotherapy does have advantages that traditional anti-tumor treatments cannot match, which can improve the prognosis of HCC patients [6, 5]. ICI therapy targeting anti-PD-1 or PD-L1 is a crucial step in a combination regimen to improve the prognosis of HCC patients [52]. The combination of anti-CTLA4 and anti-PD-L1 increased tumor-infiltrating lymphocyte function and restored HCC-derived T cell responses to tumor antigens [53]. In the present study, a novel cuproptosis-based signature was built to investigate the relationship between ICIs and the risk score as a predictor of immunotherapy response. The expression of PD-1, PD-L1, and CTLA4 was significantly lower in the high-risk group, suggesting that the signature might be potentially useful for predicting responses to ICI-targeted therapy. We observed lower TIDE scores in low-risk patients compared with high-risk HCC patients. This suggests that low-risk patients are less likely to have tumor immune evasion and are more likely to benefit from immunotherapy, which further explains their better prognosis. We next investigated the correlation between risk score and chemotherapeutic drug sensitivity. And the results indicated patients with high-risk scores seemed to be more responsive to bleomycin, dasatinib, and veliparib, while low-risk patients were more sensitive to sorafenib, lapatinib, metformin, temsirolimus, and palbociclib. The combination of immunotherapy and chemotherapy may provide precise and individualized treatment with different risk scores.

## Conclusion

We successfully identified three distinct subtypes of cuproptosis and established a novel prognostic model, providing new insights into the prediction of the outcome of HCC and its response to chemotherapy and immunotherapy.

## Data Availability

Publicly available datasets were analyzed in this study. This data can be found here: The public datasets were obtained from TCGA (https://portal.gdc.cancer.gov/) and GEO (https://www.ncbi.nlm.nih.gov/geo/).

## Abbreviations

CRGs, Cuproptosis-related genes; DCA, Decision curve analysis; DEGs, Differentially expressed genes; ICIs, Immune checkpoint inhibitors; OS, Overall survival; PCA, Principal component analysis; RCD, Regulated cell death; TCGA, The Cancer Genome Atlas; TIICs, Tumour immune infiltration cells; TME, Tumour microenvironment.
